# Culturing Layer-Specific Neocortical Neurons as a Cell Replacement Therapy Following Traumatic Brain Injury

**DOI:** 10.3389/fneur.2013.00213

**Published:** 2014-01-07

**Authors:** Nathan Peter Cramer, Mitali Chatterjee, Fritz Walter Lischka, Sharon L. Juliano

**Affiliations:** ^1^Center for Neuroscience and Regenerative Medicine, Uniformed Services University of the Health Sciences, Bethesda, MD, USA; ^2^Department of Anatomy, Physiology and Genetics, Uniformed Services University of the Health Sciences, Bethesda, MD, USA; ^3^Program in Neuroscience, Uniformed Services University of the Health Sciences, Bethesda, MD, USA

**Keywords:** traumatic brain injury, transplantation, neocortex, neuroprogenitor, therapy

## Abstract

Neurophysiological changes resulting from traumatic brain injury (TBI) can result in adverse changes in behavior including mood instability and cognitive dysfunction. Cell death following TBI likely contributes to these altered behaviors and remains an elusive but attractive target for therapies aiming at functional recovery. Previously we demonstrated that neural progenitor cells derived from embryonic rats can be transplanted into donor neonatal rat brain slices and, over the course of 2 weeks in culture, mature into neurons that express neuronal immunohistochemical markers and develop electrophysiological profiles consistent with excitatory and inhibitory interneurons. Here we examine the potential of generating electrophysiologically mature neurons with a layer-specific phenotype as a next step in developing a therapy designed to rebuild a damaged cortical column with the functionally appropriate neuronal subtypes. Preliminary results suggest that neurons derived from passaged neurospheres and grown in dissociated cell culture develop GABAergic and presumed glutamatergic phenotypes and that the percentage of GABAergic cells increases as a function of passage. After 2 weeks in culture, the neurons have a mix of immature and mature neuronal electrophysiological profiles and receive synaptic inputs from surrounding neurons. Subsets of cells expressing neuron specific markers also express layer-specific markers such as Cux1, ER81, and RORβ. Future studies will investigate the potential of transplanting layer-specific neurons generated and isolated *in vitro* into the neocortex of neonatal brain slices and their potential to maintain their phenotype and integrate into the host tissue.

## Introduction

Traumatic brain injury (TBI) results in a host of adverse changes within the central nervous system that can have profound adverse functional and behavioral changes. While the underlying pathology of these deficits is still under investigation, the loss of neural tissue from initial impact or secondary injury mechanisms contributes to these deficits (1). The use of stem cells to replace lost neurons or rebuild missing networks holds great promise for reversing TBI-induced neurological deficits (2, 3). However, our ability to rebuild damaged neuronal networks is limited in part by our capacity to generate specific types of neurons in a controlled fashion. *In vitro* methods that successfully generate neurons phenotypic of the different neocortical layers may help bring viable cell replacement therapies closer to reality.

Within the neocortex, neurons that ultimately reside within a particular cortical layer are born in the ventricular and subventricular zones (VZ and SVZ) arising from neural progenitor cells in both these regions (4–8). Neurons of the deeper cortical layers are born first and subsequent superficial layers arise from later born neurons resulting in an inside-out construction pattern (9). Thus, neurons of the different cortical layers are generated from different cell division cycles of progenitor cells in the VZ and SVZ. These observations suggest that the number of cell division cycles could be a contributing factor to the final neuronal subtype (10–12) and replication of this process *in vitro* might be capable of generating neurons phenotypic of specific cortical layers. Shen et al. (13) on the other hand, demonstrated that neural progenitor cells harvested from embryonic mice at the onset of corticogenesis (E10.5) contain the machinery for producing multiple neocortical cell types when grown in culture.

As a potential source for a viable mixed population of neurons capable of transplantation we investigated the capacity for neuroprogenitor cells (NPC) harvested from the neocortex of later stage mouse embryos (embryonic days 14–16) to generate neurons characteristic of different cortical layers when grown in culture long enough to become electrophysiologically mature. Furthermore, we investigated whether propagation of these cells through repeated passaging as neurospheres affected the overall composition of subsequent cultures once allowed to differentiate for multiple weeks. Finally, we investigated the ability of later stage embryonic neurons to maintain neuronal identities and integrate with host tissue when transplanted into injured slices of neonatal mouse cortex. We find that this approach successfully generates electrophysiologically mature neurons that express markers characteristic of different neocortical layers and that acutely isolated cells successfully integrate into neuronal networks upon transplantation. These studies help further our understanding of the potential for NPC to serve as a therapeutic source for treating TBI.

## Materials and Methods

### Harvesting of embryonic cortical cells

Pregnant wild type C57Bl/6 or C57BL/6-Tg(UBC-GFP)30Scha/J mouse dams from Jackson Laboratories were injected with euthasol (~0.1 cm^3^ per 15 g body weight, IP) and embryos (E14–16) removed under sterile surgical conditions. The developing sensory cortex was isolated from each embryo while submerged in sterile ice-cold artificial cerebrospinal fluid continuously bubbled with 95/5% O_2_/CO_2_. Single cell suspensions were generated by mechanical trituration. Cells were then either used for neurosphere/cell culture experiments or injection into neonatal mouse slice cultures as outlined below.

### Generation of cortical slices from neonatal mice

WT mouse pups (P1–P3) were heavily anesthetized with isoflurane and decapitated with sharp scissors. The brains were removed and 400 μm thick coronal hemi-sections were cut in sterile filtered ice-cold artificial cerebrospinal fluid continuously bubbled with 95/5% O_2_/CO_2_. Sections were transferred to sterile tissue culture inserts in a six well plate (one to four sections per well) and washed twice with sterile MEM containing 10% normal horse serum and 4% G:1:2. TBIs were simulated by making a fine incision in the cortex with a 22 gauge needle. A total of 30,000 harvested GFP^+^ embryonic cells were injected into each incision in a volume of 10 nl culture medium. Slices were incubated for 1–2 weeks prior to immunohistochemistry or electrophysiology experiments.

### Neurosphere passaging and differentiation

Harvested embryonic neurons not used for injection were maintained in proliferation media (NeuroCult NSC Basal Medium Mouse, STEMCELL Technologies Inc., Vancouver, BC, Canada) until neurospheres formed. Upon reaching sufficient size, neurospheres were partially dissociated with brief treatment of trypsin (Gibco 0.05%Trypsin EDTA), washed, and divided into two groups. One group was placed in new proliferation media for a subsequent passage and the other used for cell culture experiments. For cell cultures, dissociated neurospheres were plated on coverslips at a density of 6e10 cells per milliliter and maintained in differentiation media (NeuroCult NSC Basal medium Mouse with Proliferation supplement, STEMCELL Technologies Inc., Vancouver, BC, Canada) media for 1–2 weeks prior to use for immunohistochemistry or electrophysiological experiments.

### Electrophysiology

Slices with GFP^+^ transplanted cells or coverslips with cultured cells were transferred to a submersion style chamber attached to a Zeiss Axioskop; individual neurons were visualized with DIC optics. GFP^+^ transplanted neurons were identified by their fluorescence. Slices and cover slips were continuously perfused with ACSF composed of (in millimolar) NaCl 126, KCl 3, CaCl_2_ 2, NaH_2_PO_4_ 1.25, MgSO_4_ 2, NaHCO_3_ 26, d-glucose 10, bubbled with a mixture of 95% O_2_/5% CO_2_ at room temperature. Whole cell recordings were obtained with borosilicate pipettes containing (in millimolar): K-gluconate 130, KCl 15, HEPES 5, EGTA 1, Mg-ATP 4, Na-GTP 0.3 with pH adjusted to ~7.3 with KOH. In a subset of recordings, 0.2% Neurobiotin was included in the intracellular solution to enable *post hoc* visualization of recorded cells (Vector Laboratories, Burlingame, CA, USA). Analysis of active and passive membrane properties was performed offline using ClampFit software (Molecular Devices, LLC, Sunnyvale, CA, USA) and custom written routines in Igor Pro (WaveMetrics, Portland, OR, USA). Spontaneous synaptic activity was analyzed by isolating individual synaptic events in MiniAnalysis (SynaptoSoft, Decatur, GA, USA).

### Immunohistochemistry

Coverslips with dissociated cell cultures were fixed with 4% paraformaldehyde followed by four washes with PBS. After a 1 h incubation in blocking buffer at room temperature, coverslips were incubated in blocking buffer plus either mouse monoclonal anti-β-tubulin (1:500), rabbit polyclonal anti-MAP-2 (1:200, Sigma Aldrich, St. Louis, MO, USA), or mouse monoclonal anti-NeuN (1:100, EMD Milliporem, Billerica, MA, USA) and a layer-specific (rabbit anti-ER81, 1:1000; mouse anti-CUX1, 1:1000; rabbit anti-RORβ, 1:500; abcam, Cambridge, MA, USA) or rabbit anti-GABA (Sigma Aldrich, St. Louis, MO, USA) overnight at 4°C. Coverslips were washed in PBS and a secondary antibody (Alexa Fluor 488 or 546, 1:200; Life Technologies, Grand Island, NY, USA) applied for 90 min followed by a final rinse in PBS and application of bisbenzimide to label cell nuclei. Finally coverslips were mounted on glass slides using Mowiol or Vectashield. Slice cultures were fixed in 4% paraformaldehyde for 20 min followed by overnight incubation in a 30% sucrose/PBS (wt/vol) solution. Slices were sub-sectioned into 20–40 μm thick sections for subsequent immunohistochemistry that proceeded as above following permeabilization with 0.1% Triton X-100 in PBS. All antibody concentrations were the same except anti-β-tubulin (1:1000) and secondary antibodies (1:1000). Neurobiotin filled cells were visualized by incubating either cell or tissue cultures with NeutrAvidin-488 (Life Technologies Corporation) diluted 1:200 in PBS for 2 h following primary and secondary antibody reactions.

Images were imported into Fiji (14) and each acquisition channel separated into individual images. The channel containing data from a neuronal marker (NeuN, β-tubulin, or MAP-2) was thresholded using Otsu or Maximum Entropy auto-thresholding. These parameters reliably isolated cell bodies from the background. Regions of interest (ROIs) were drawn around individual neurons and the resulting series of ROIs overlaid on the similarly thresholded image containing a layer-specific marker (Cux1, ER81, or RORβ) or GABA imaging data. The percentage of the neuronal marker ROI filled with a layer-specific or GABA pixels was calculated and a value of greater than 30% of the neuronal ROI was considered co-labeled. DAPI stained nuclei were hand counted using the Cell Counter plugin.

## Results

### Neurogenic potential and expression of layer-specific markers

Neuroprogenitor cells capable of generating multiple subtypes of neurons are a potential source for restorative therapies following TBI. We investigated whether cells isolated from the neocortex of embryonic mice during the peak period of corticogenesis [E14–16 (15, 16)] could produce and sustain neurons when cultured as neurospheres and whether the composition of neuronal subtypes changed over repeated passages. After ~7 days in culture, neurospheres were either placed in differentiation media (see Materials and Methods) and allowed to differentiate for at least 2 weeks or back in proliferation media. This was repeated for up to 3 passages. When neurons were differentiated, they were immunoreacted for neuronal markers, laminar markers, and GABA. Results were similar across embryonic ages and were consequently combined.

**Figure 1:**
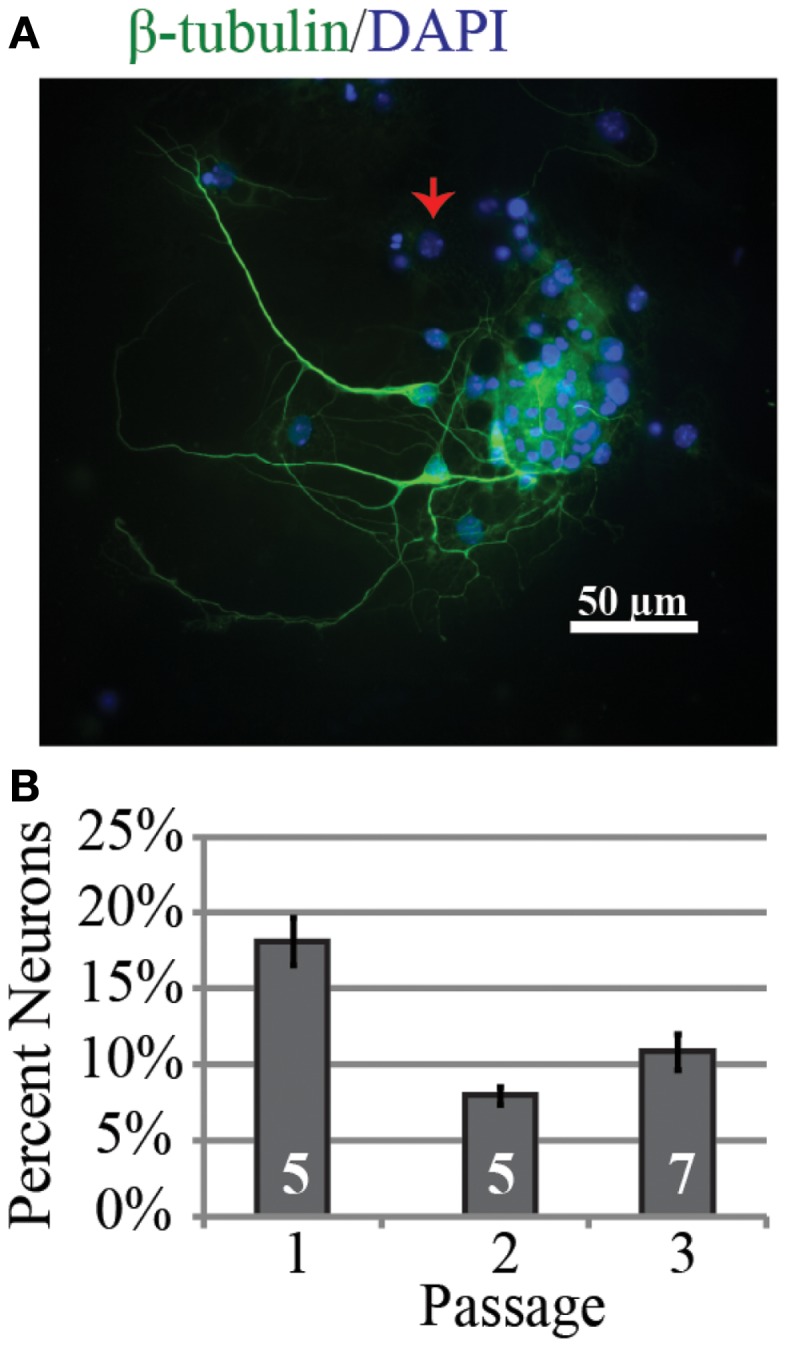
**Neurons can be successfully generated from passaged neurospheres even after multiple passages**. **(A)** Representative example of neurons from passage 3 after 9 days *in vitro* immunopositive for the neuronal marker β-tubulin (green) and DAPI labeled cell nuclei (blue). The red arrow indicates a nucleus from a β-tubulin negative cell. **(B)** Group data for percentage of neurons identified as a function of neurosphere passage number. The percentage was highest in passage 1.

**Figure 2:**
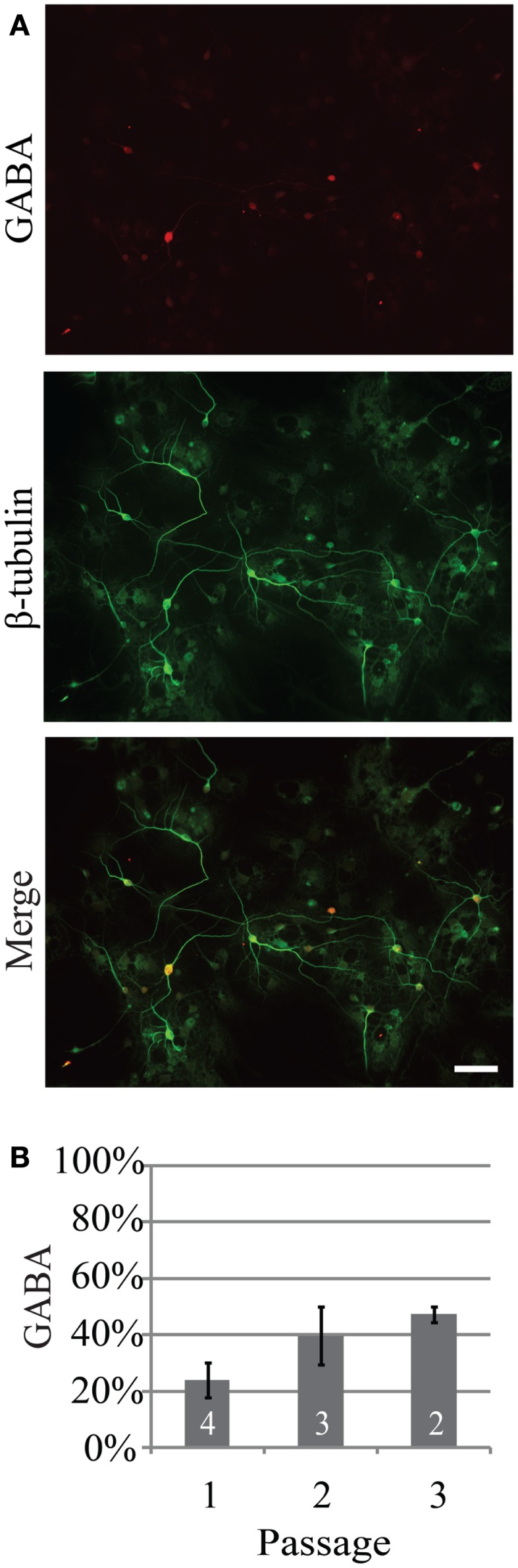
**The percentage of neurons generated from neurospheres that are GABAergic increases as a function of passage**. **(A)** Representative example of GABAergic immunoreactivity (red) in neurons from passage 2, 14 days *in vitro*. Neurons were identified by expression of β-tubulin (green). **(B)** Percentage of neurons that were GABAergic following three consecutive passages. Scale bar equals 50 μm.

Investigation of the neurogenic potential of cells following passaging revealed that each passage was capable of generating neurons (Figure 1). Passage 1 generated the highest percentage of neurons (18 ± 6%; *n* = 723 cells, five experiments) compared to 9 ± 2% (*n* = 301 cells, five experiments) and 11 ± 4% (*n* = 294 cells, seven experiments) for passages 2 and 3 respectively. This pattern is similar to that reported for NPC harvested from e10 embryos (13). In addition to the development of presumed glutamatergic projection neurons we examined the development profile of GABAergic neurons across passages (Figure 2). The percentage of neurons immunopositive for GABA in passage 1 was 24 ± 6% (*n* = 240 neurons from four experiments) compared to 40 ± 10% (*n* = 102 neurons from three experiment) and 47 ± 3% (*n* = 59 neurons from two experiments) in passages 2 and 3 respectively (Figure 2B). Thus the percentage of inhibitory neurons tended to increase as a function of passage number although the difference was not significant (*P* = 0.34, ANOVA).

**Figure 3:**
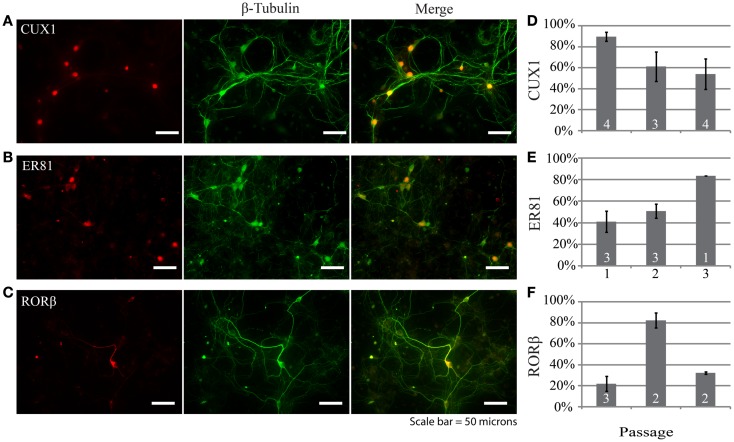
**Neurons generated by differentiating dissociated neurospheres express markers characteristic of different neocortical layers such as CUX1 (A), ER81 (B), and RORβ (C)**. In each example the layer-specific marker is indicated in red and the neuronal marker β-tubulin in green. **(D–F)** Group data depicting the distribution of marker expression across neurospheres passages one through three. Scale bar equals 50 μm.

Examination of the layer specificity of neurons derived from passaged neurospheres and allowed to differentiate for multiple weeks *in vitro* revealed a diverse set of neuronal phenotypes. Figures 3A–C shows representative examples of neurons differentiated from neurospheres. In this example, a marker for upper layer neurons, Cux1 (Figure 3A), colocalizes with a subset of cells immunopositive for a neuron specific marker (β-tubulin, green). In passage 1, expression of Cux1 was highest (Figure 3D, 89 ± 4%, *n* = 161 cells from four experiments) while the fraction of neurons expressing Er81, a marker of earlier born deeper layer neurons (17, 18), was lowest (Figures 3E, 40 ± 10%, *n* = 100 cells from three experiments). RORβ expression levels, a marker of layer IV neurons (18), was in between these two levels at 56 ± 4% (Figure 3F, *n* = 212 cells from three experiments). Thus, neurons characteristic of multiple layers of the neocortex were present but upper layer neurons were dominant.

We also investigated whether repeated passaging of neurospheres derived from E14 to E16 embryos altered the neuronal composition of cultures following differentiation. In general, there was variability between passages, but markers for all layers were present in each passage. The distributions did not follow a specific pattern, but markers for all layers were substantially represented after each passage. The persistence of neurons immunoreactive for Er81 suggests that these cells may survive better than those expressing markers for layer 4 (RORβ) or the upper layers (Cux1) (Figures 3D–E). These findings suggest that NPCs obtained from proliferative regions at E14–16 are capable of generating neurons intended to reside in multiple layers; this is not surprising given what is known about the dates of generation of mouse neocortical neurons (10–12). Cells expressing GABA also increased in percentage with passage number. This suggests that GABAergic cells may also be more likely to survive over a series of passages in culture perhaps through reduced induction of apoptotic mechanisms as seen in neonatal rat cerebellar cultures (19).

### Electrophysiological properties of cultured neurons

We examined the electrophysiological properties of the cultured neurons using whole cell recordings. Neurons from passage 2 tended to have higher membrane resistances compared to the other passages (757 ± 108, 985 ± 169, 798 ± 105 MΩ for passages 1, 2, and 3 respectively, Table 1) however the difference was not significant (*P* = 0.83). There was no significant difference in either the membrane capacitance or resting potential across passages (Table 1). Passage 1 cells had a higher current threshold for initiation of action potentials (85 ± 10 pA) compared to passage 2 and 3 (51 ± 7 and 61 ± 10 pF respectively). This difference was significant for passage 2 (*P* < 0.03, ANOVA with Tukey’s *post hoc* comparison, Table 2).

**Table 1:** **Passive membrane properties of neurons differentiated from neurospheres**.

	Psg	Mean	SEM	*N*	*P*
Membrane resistance (MΩ)	1	757	108	30	0.83
	2	985	169	20	
	3	798	105	12	
Membrane capacitance (pF)	1	39	3	30	0.82
	2	34	3	20	
	3	36	5	12	
Resting potential (mV)	1	−56	1	30	0.85
	2	−58	2	20	
	3	−57	2	12	

**Table 2:** **Active membrane properties of neurons differentiated from neurospheres**.

	Psg	Mean	SEM	*N*	*P*
Rheobase (pA)	1	85	10	27	0.03
	2	51	7	16	
	3	61	10	12	
Threshold (mV)	1	−32	1	27	0.77
	2	−32	2	16	
	3	−33	1	12	
HWHM (ms)	1	1.6	0.1	27	0.78
	2	1.5	0.1	16	
	3	1.6	0.2	12	
Peak rise rate (mV/s)	1	55	6	27	0.83
	2	58	7	16	
	3	52	5	12	
Peak decay rate (mV/s)	1	−21	2	27	0.78
	2	−22	2	16	
	3	−23	2	12	

Neurons from passage 2 had a lower action potential threshold than those from passage 1 (*P* = 0.03 ANOVA with Tukey’s *post hoc* comparison).

**Figure 4:**
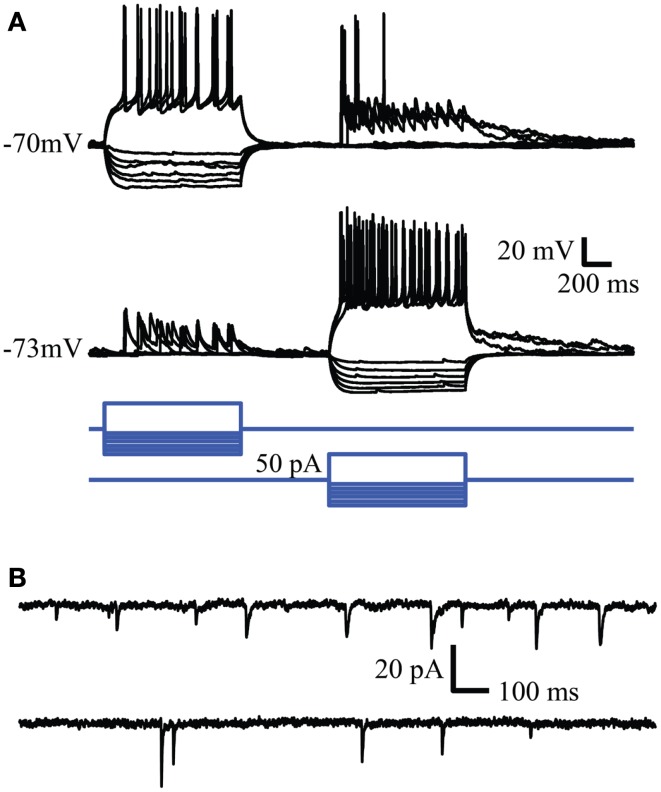
**Whole cell recordings reveal synaptic connectivity between neurons in culture**. **(A)** Reciprocal connections in cultured neurons. Action potentials evoked in one neuron evoke EPSPs in the simultaneously recorded second neuron. **(B)** Spontaneous synaptic activity recorded in the same cells. Similar synaptic activity was observed in single cell recordings.

Spontaneous synaptic activity between neurons tended to decline as a function of passage number. The number of quiet cells was lowest in passage 1 (4 out of 28, 14%) and highest in passage 3 (7 out of 12, 58%). In passage 2, 4 out of 20 cells were silent (20%). The frequency of synaptic inputs tended to be higher in passage 2 (0.5 ± 0.2 Hz vs. 0.32 ± 0.07 and 0.3 ± 0.2 Hz for passages 1 and 3 respectively) but the difference was not significant (*P* = 0.19). Examples of evoked and spontaneous synaptic activity are shown in Figures 4A,B respectively. In Figure 4A simultaneous whole cell recordings were used to evoke action potentials in one cell (square steps in the blue command traces) while recording the resulting synaptic activity in the other cell. In contrast to action potential mediated responses, hyperpolarizing each cell did not produce a change in the opposite cell suggesting the absence of gap junctions between the two cells. Voltage clamp recordings of spontaneous postsynaptic currents (PSC) activity for these cells is shown in Figure 4B and is representative of activity observed in all neurons with active inputs. Bath application of 500 nM gabazine, a GABA_A_ receptor antagonist, changed the frequency of synaptic activity but in a variable manner between cells (data not shown) suggesting the presence of functional GABAergic synapses within the cultured network.

**Figure 5:**
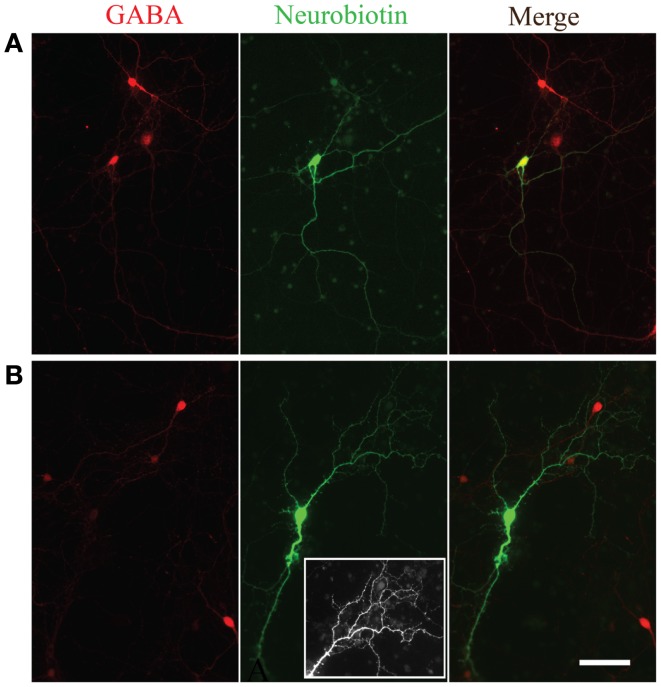
**Morphological evidence for GABAergic and glutamatergic neurons**. GABA immunoreactivity (red, left panels) in two neurons filled with Neurobiotin (green, middle panels). The overlay of the two channels is shown in the right panels. Note the smooth appearance of processes from the neuron immunopositive for GABA **(A)** compared to the spiny appearance of the cell immuno-negative for GABA **(B)**. Inset in the bottom middle panel is an expanded view of the spiny processes on the dendritic tree of the neuron immuno-negative for GABA.

In a subset of recordings, Neurobiotin was included in the intracellular solution. Figure 5 shows two examples of Neurobiotin labeled cells following immunohistochemistry for GABA. The cell in Figure 5A was positive for GABA (upper left) and had smooth processes relatively free of spines, a morphology consistent with neocortical inhibitory neurons (20). Conversely, the neuron depicted in the Figure 5B was immuno-negative for GABA (lower left) and, like excitatory neurons of the neocortex (21), had many visible spinous processes when visualized with NeutrAvidin-Alexa488 (lower middle panel; inset shows closer view of dendritic tree). Although neurons across passages were capable of firing multiple action potentials, we did not observe the distinct fast-spiking firing pattern of GABAergic neurons observed in slices of the neocortex or *in vivo* (22–24).

### Transplantation of embryonic neurons

**Figure 6:**
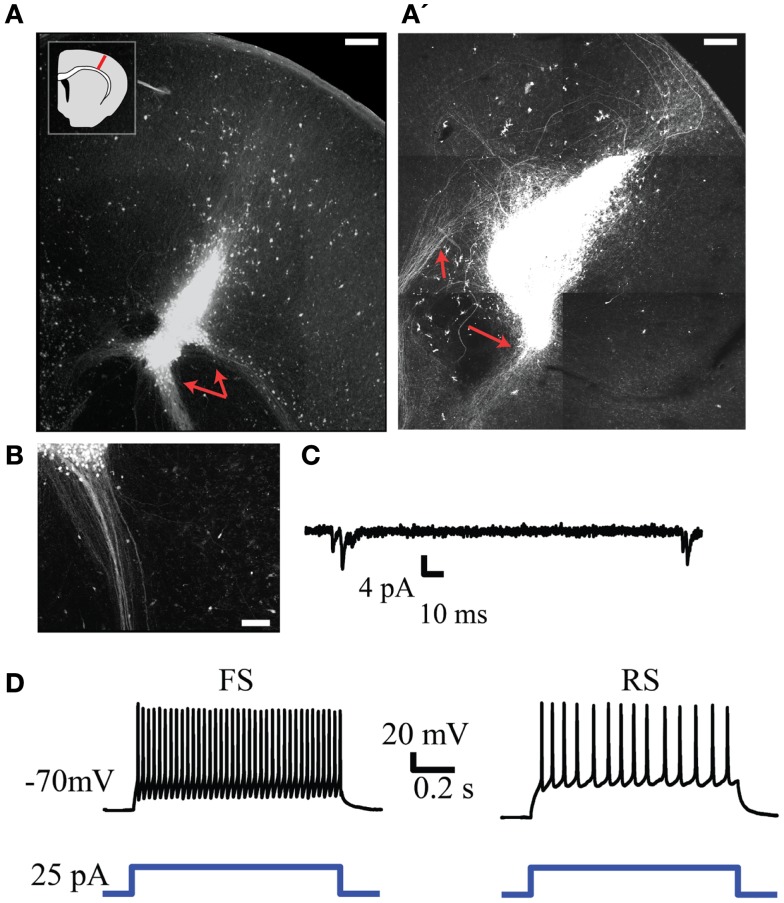
**Injection of GFP^+^ NPCs into lesions within neonatal embryonic neocortex results in viable neuronal transplants**. **(A,A’)** Representative examples of an injury site 7 days after injection of GFP^+^ NPCs into the lesion. Subcortically projecting GFP^+^ fibers arising from the transplant are indicated with red arrows. The inset in **(A)** depicts the approximate region of injury within each slice. **(B)** Presumed axons arising from the transplant tended to form dense projections when projecting subcortically. **(C)** Example of spontaneous synaptic activity in a transplanted GFP^+^ neuron. **(D)** Firing patterns typical of fast spiking (FS, left) and regular-spiking (RS, right) neurons recorded from transplanted cells. Bottom trace depicts the depolarizing current command. Scale bars in **(A,A’)** are 250 and 100 μm in**(B)**.

To investigate the regenerative potential of NPC we harvested cells from the neocortex of embryonic GFP-expressing mice and transplanted them into an injured region in the neocortex of brain slices obtained from neonatal mice. GFP-expressing cells remained largely confined to the injury site and frequently retained or established morphologies consistent with neurons. The transplanted cells were also capable of generating long range neurite projections that presumably consisted of axons (representative examples in Figure 6). These projections tended to be most dense and focused when projecting away from the cortex (red arrows in Figures 6A,B) and more diffuse within the neocortex (Figure 6A’). Electrophysiological recordings from GFP^+^ cells 4–7 days after transplantation displayed neuronal phenotypes with spontaneous synaptic inputs present in four out of eight cells (representative example in Figure 6C, mean frequency of 0.12 ± 0.4 Hz). GFP^+^ neurons also exhibited firing patterns typical of fast spiking and regular-spiking neurons in response to depolarizing current steps (presumed inhibitory and excitatory neurons respectively, Figure 6D). The electrophysiological properties of the transplanted neurons did not differ over the time points examined.

## Discussion

Damage to the neocortex either through TBI or focal hypoxia can lead to destruction of impacted regions of the neocortex and can result in behavioral deficits. These deficits can be severe and significantly impact the quality of life in affected individuals. With the increased use of improvised explosive devices in the wars in Afghanistan and Iraq there has been a sharp rise in individuals suffering from the effects of TBI (25, 26). However, our ability to treat and reverse the behavioral deficits resulting from brain trauma remains limited. The ability to replace lost or damaged neocortical networks through transplantation of NPC may result in functional recovery from the injury (27). The goal of our present study was to investigate how donor NPC could be guided toward becoming neurons phenotypic of a specific cortical layer as part of a targeted cell replacement therapy. Specifically, we hypothesized that reproducing *in vitro* the iterative cell division cycle that occurs in the developing neocortex during embryogenesis *in vivo* would generate specific neuronal subtypes.

In mice, the neurons populating the neocortex are generated during embryonic days 11 through 17 (E11 and E17 respectively) from the ventricular and subventricular zone (11). Located on the ventricular walls of the developing cortex, NPC repeatedly divide giving rise to neurons that migrate dorsally through previously populated layers of the neocortex resulting in an inside-out manner of construction [e.g., (4, 28–30) for reviews]. NPCs undergo 11 cycles of division with a duration that increases with subsequent divisions (11). Thus, in addition to other trophic signaling factors in the extracellular milieu, the duration and number of cell division cycles appears to play a role in determining the final fate of newly born neurons during embryonic development (12). Upon completion of neocortical development, neurogenesis in the proliferative regions of the cerebral cortex largely ceases (31) while patterning of neuronal connectivity continues. Although injury to the brain can stimulate some degree of neurogenesis from NPCs present in the mature brain (32–35), this apparent attempt at circuit repair is insufficient for restoring damaged networks.

As a potential avenue for overcoming the limitations to repair damaged neocortical networks, we sought to proliferate layer-specific neurons *in vitro* using relatively developed embryonic mouse neocortex as a tissue source. We found neurons expressing layer-specific markers (Cux1, RoRβ, and Er81) were present following a single cycle of neurosphere proliferation and differentiation. Additionally, although the relative composition changed, neurons with these markers remained present through the last passage examined, passage 3. Thus this process is capable of producing neurons phenotypic of upper, middle, and deep cortical lamina (17, 18, 36). Our results also support the hypothesis that the mechanisms required to guide neuronal fates are at least partially intrinsic to the cells themselves (13).

In addition to presumed excitatory layer-specific neurons we found that repeated passaging of neurospheres generated from embryonic mouse neocortex also produced GABAergic neurons. The percentage of cells immunopositive for GABA increased as a function of passage. The ability to generate GABAergic neurons is essential for maintaining the proper balance between excitation and inhibition with any putative transplant. Excessive excitatory drive within a cortical network may lead to seizure activity and exacerbation of damage to the neocortex.

Electrophysiological recordings from cells differentiated from neurospheres revealed active and passive membrane properties consistent with neurons. Recorded cells had hyperpolarized resting potentials and membrane resistances and capacitances consistent with neurons. The individual values varied widely between different neurons representing a range of maturity similar to those observed in neurons generated from an mouse ES cell line (37, 38). These differences likely reflect a lower density of ion channels in the cell membrane and a less expansive dendritic tree compared to more mature neurons. Both mature and immature-like neurons were present in all three passages examined suggesting similar maturation rates of neurons from each passage. When acutely isolated cells from embryonic mouse neocortex were transplanted into neonatal brain slices we observed a similar development and maturation of neurons from the transplant. Although the phenotype of the neurons was not examined immunohistochemically after transplantation into a slice, we observed neurons with fast- and regular-spiking activity characteristic of inhibitory and excitatory neurons respectively. We also observed that neurons grown either in dissociated cell cultures from neurospheres or in brain slices developed functional synaptic contacts. These results suggest that transplanted neurons can indeed develop and maintain neuronal properties and successfully integrate with surrounding neurons.

As these and similar studies progress it will be important to consider the role of glial cells that develop from the transplanted population. The formation of glial scars following CNS injury are associated with a diminished capacity for endogenous rebuilding of damaged neuronal networks (39, 40). Thus, although we have previously demonstrated that glia are rare in similar neural progenitor cell cultures (41), glia arising from transplanted cells may potentially contribute to scar formation and or, conversely, they may produce supportive substances (42–44) that encourage the health of transplanted cells. Although we did not directly investigate whether the presumed axons we observed following transplantation were myelinated, similar studies have found that oligodendrocytes arise from grafted neural stem or progenitor cells (45, 46). Thus transplanted NPCs that become glia may contribute to the overall health of the transplant and its functional integration with the host networks. Isolating layer-specific neurons generated *in vitro* either through successive passaging as we and others describe (13), or actively through the regulation of cell signaling pathways (47–49) could help achieve the ideal balance between neurons and supportive glia within the transplant. An additional challenge will be successfully transplanting cells into adult tissue where the environment is less conducive to transplant survival.

In this work we investigated the potential of generating layer-specific neurons *in vitro* for potential transplantation into damaged regions of the neocortex. We found that repeatedly passaging neurospheres and differentiating the resulting cells can produce a range of neurons characteristic of those found in the mature neocortex both in electrophysiological characteristics and in the expression of layer-specific markers. Acutely isolated NPCs transplanted into neonatal brain slices could develop into electrophysiologically mature neurons and form functional synaptic connections with the surrounding neurons. Together, our results suggest that generation of specific neocortical neurons *in vitro* may lead to viable network reconstruction therapies to restore lost or damaged neuronal networks.

## Author Contributions

Nathan Peter Cramer designed the experiments and performed cell transplants, slice cultures, electrophysiological recordings, collected and analyzed the data, and prepared the manuscript. Mitali Chatterjee harvested embryonic tissue, maintained neurosphere cultures, performed immunohistochemistry, and assisted with image collection. Fritz Lischka performed electrophysiological recordings. Sharon L. Juliano designed the experiments, oversaw all aspects of data collection and analysis and prepared the manuscript.

## Conflict of Interest Statement

The authors declare that the research was conducted in the absence of any commercial or financial relationships that could be construed as a potential conflict of interest.
